# Community violence in neighborhoods and common mental disorders among Brazilian adolescents

**DOI:** 10.1186/s12888-023-05269-8

**Published:** 2023-10-23

**Authors:** Claudia Reis Miliauskas, Washington Junger, Natalia Hellwig, Katia Vergetti Bloch, Claudia de Souza Lopes

**Affiliations:** 1 Medical Sciences College, Department of Medical Specialties, State University of Rio de Janeiro, Vinte E Oito de Setembro Avenue, 77, 4 Floor, 432. Vila Isabel, Rio de Janeiro, 20.551-030 Brazil; 2 Institute of Social Medicine, State University of Rio de Janeiro, São Francisco Xavier Street, 524, Maracanã, 7 Floor, Rio de Janeiro, 20.550-013 Brazil; 3 Institute of Studies in Public Health at Universidade Federal Do Rio de Janeiro (IESC), Horácio de Macedo Avenue, no number, Ilha Do Fundão, Rio de Janeiro, 21941-598 Brazil

**Keywords:** Mesh terms, Violence, Adolescent, Mental health. no mesh terms, Community violence, Common mental disorders

## Abstract

**Background:**

This study aims to explore the association between exposure to neighborhood violence and the presence of common mental disorders (CMDs) among Brazilian adolescents aged 12 to 17 years. Additionally, we aim to analyze whether sex, age and race are modifiers of the effect of this association.

**Methods:**

The study comprised 1,686 adolescents residing in the municipality of Rio de Janeiro, situated in the Southeast region of Brazil. To measure exposure to community violence, we constructed three crime indicators using data from Civil Police of the State of Rio de Janeiro: crimes against property, nonlethal crimes, and lethal crimes. Employing geospatial analysis based on the adolescents’ residence location, logistic regression modeling was performed to measure the association between violence and CMDs.

**Results:**

Adolescents living in regions with higher rates of the three types of violence studied herein were more likely to have CMDs, with odds ratios (ORs) ranging from 2.33 to 2.99. When stratified by sex, age and race, girls, older adolescents and blacks have a greater magnitude of effect on the measure of association, indicating a heightened risk for CMDs.

**Conclusion:**

This study provides important contributions to the public health field, as it reveals new information on the influence of community violence on the mental health of adolescents. Given the elevated rates of violence globally, knowing the effects of such violence on adolescents becomes crucial for the prevention and treatment of CMDs within this population.

## Introduction

Common mental disorders (CMDs) correspond to a group of symptoms encompassing anxiety, depression, and somatic complaints, though not necessarily indicative of pathologies [1]. CMDs are highly prevalent in the general population. A study carried out among Brazilian adolescents showed a prevalence of CMDs equal to 30%, with a higher proportion among girls (38.4%) and older adolescents (36.6%) [2]. In adolescents, such symptoms disrupt school life and family and social relationships and are highly persistent [3, 4].

Identifying risk and protective factors for CMDs in adolescents is of paramount importance. Community violence (CV) has been shown to be an important risk factor for mental disorders in adolescents [5] and can be defined as violence between individuals without a personal relationship who may or may not know each other, occurring outside the home [6]. Among the various forms of CV, homicide stands as one of the most grievous; statistics from the year 2000 estimated 199,000 homicides among young individuals globally, translating to a rate of 9.2/100,000 inhabitants. Homicide rates display substantial regional disparities, with the highest incidences observed in Latin American countries (84.4/1,000,000 in Colombia and 30.2/100,000 in Brazil) and the lowest rates observed in European countries (0.6/100,000 in France and 0.9/100,000 in England) and Asian countries (0.4/100,000 in Japan). Notably, across all countries, males are disproportionately more susceptible to becoming victims of homicide than females [7].

The influence of CV on the mental health of adolescents has been the subject of two meta-analyses and three reviews recently [5, 8–11]. A clear relationship exists between increased exposure to CV and the development of mental disorders, including CMDs; however, the factors that may influence this relationship are not yet clear. The female gender appears to be a risk factor for internalizing disorders, with girls demonstrating a heightened likelihood of symptoms of depression and anxiety than boys under equivalent exposure to violence. Additionally, factors such as race and age appear to exert an influence in this relationship. However, only a limited number of studies have undertaken an assessment of these variables as potential effect modifiers within this association [11].

CMDs are more prevalent among girls than boys [12]. One plausible factor contributing to this difference may be the impact of the sexist culture during the upbringing of children and adolescents. Boys, from an early age, are often encouraged to suppress their emotions and avoid displaying vulnerability, commonly interpreted as a sign of weakness. At the same time, they are encouraged to occasionally exhibit aggressive behavior among their peers and engage in activities such as substance use and early sexual experimentation. Girls, on the other hand, are typically encouraged to adopt caretaker roles within the family and home, with emphasis on avoiding conflicts and confrontations, among other protective behaviors. Existing literature shows that externalizing disorders, such as conduct disorder, substance use disorders and disruptive behaviors, are more prevalent in boys, whereas internalizing disorders, such as CMDs, depression and anxiety, are more commonly observed in girls [2, 13, 14]. Drawing from this knowledge and operating under the assumption that a culture of traditional masculinity influences girls to manifest a greater degree of depressive and anxious symptoms than boys, we hypothesize that they are at an elevated chance of developing CMDs than boys when exposed to community violence.

Regarding age, it is remarkable that older adolescents often engage in greater social interactions and consequently encounter higher levels of exposure to community violence (CV). Conversely, some literature hypothesizes that they might also possess a greater degree of emotional maturity and a more extensive array of psychological resources for effectively coping with adversities, hence the findings within the literature vary [11]. Considering CV exposure as an event with a high emotional impact, we posit that older adolescents are more likely to develop CMDs compared to younger adolescents. This hypothesis is rooted in the notion that the heightened emotional impact of such exposure could potentially render older adolescents more susceptible to the onset of CMDs.

Regarding race, a meta-analysis investigated the relationship between racism and physical and mental health and revealed a significant association between experiences of racism and poorer mental as well as physical health [15]. However, as presented by Oliveira [16], the examination of race remains among the most underexplored factors within health indicators, thus underscoring the persistent existence of structural racism. Illustrating this issue, data from the Institute of Public Safety of the State of Rio de Janeiro for the year 2000 reported that out of 4,907 victims of violent crime, a majority of 68.8% were identified as brown or black, while only 20.2% were identified as white; information regarding race was unavailable for 11% of the victims [17]. Furthermore, as presented by Waiselfisz, reports from 2003 to 2014 showed that Brazil witnessed a decline in firearm-related homicide among white individuals, i.e., from 14.5 to 10.6 per 100,000, representing a reduction of 27.1%. In stark contrast, among black individuals, an opposing trend emerged, with rates increasing from 24.9 to 27.4 per 100,000, representing an increment of 9.9% [18]. Therefore, considering that black adolescents disproportionately bear the load of community violence and that racism distinctly detrimentally impacts their mental well-being, we posit the hypothesis that individuals of black ethnicity face a heightened likelihood of developing CMDs in comparison to white adolescents.

We justify the present study based on the high prevalence of CMDs among adolescents and the high incidence of violence occurring in communities. Both CV and CMDs are public health problems and are amenable to interventions. The literature points to a clear relationship between exposure to CV and an increased risk of CMDs, but the effects of race, sex and age on this relationship still need to be clarified. In addition, few studies have been carried out in low- and middle-income countries, where CV rates are substantially higher. We can suppose that in places with greater exposure to violence, its effect on the mental health of adolescents may be different from that in places with lower rates. Finally, few studies in the literature have used indicators of violence based on criminal statistics, since differences may exist between objective and subjective measures of violence in the association with mental disorders in adolescents. More studies need to be carried out with these indicators.

Brazil, and more specifically, the city of Rio de Janeiro, possesses social, cultural, and economic characteristics that have been largely associated with increased prevalence of mental disorders. Notably, exposure to violent communities stands out as a factor with significant impact on life quality, particularly among adolescents who are beginning to explore the environment more freely. Our hypothesis is that exposure to community violence is a significant risk factor to increased prevalence of common mental disorders among adolescents. Furthermore, we hypothesize that the older, the females, and the black skin adolescents encounter amplified risk compared to their peers. Through an examination of these associations, our study aims to demonstrate the impact of community violence on mental health and to address potential discrepancies among distinct groups within the adolescent population. With this study we also aim to provide insights for potential interventions and support in health care systems.

## Materials and methods

### Baseline study and population

This study includes a subsample from the Cardiovascular Risk Study in Adolescents (ERICA). ERICA was conducted with a random sample, with national and school representativeness, and included 74,589 adolescents aged 12 to 17 years who were students at public and private schools. The inclusion criteria for ERICA were as follows: students aged 12 and 17 years who were enrolled in public or private education between the seventh year of elementary school and the third year of high school. The exclusion criteria were as follows: students who were physically disabled and unable to perform anthropometric assessments, students who were obese and students who were pregnant. Data were collected from March 2013 to December 2014. Individual data from the adolescents were collected through a self-administered questionnaire using an electronic personal data assistant (PDA) [19].

The subsample used in this study included 1,694 adolescents living in the municipality of Rio de Janeiro, which is located in the Southeast region of Brazil. Among these adolescents, eight were lost due to a lack of information; therefore, the final sample consisted of 1,686 adolescents. The reason for selecting this subsample was the possibility of georeferencing residential addresses; this information was available only for adolescents who had blood collected for ERICA and allowed access to public safety data based on neighborhoods in the municipality where the adolescents resided. The geocoding success rate was 75%.

The study subsample comprised adolescents participating in ERICA and residing in the municipality of Rio de Janeiro, and for which we had addresses for georeferencing and data from the Public Security Institute. Therefore, the study did not encompass the entire metropolitan region of Rio de Janeiro, focusing solely on adolescents within the municipality area.

### Exposure variable

To measure CV exposure, based on the definition of CV as violence occurring outside the home and mostly by unknown people, we used three crime indicators based on the Proposed Indicators of Violent Crime^1^: (i) crimes against property, (ii) non lethal crimes, and (iii) lethal crimes. The first indicator included robbery, theft and extortion; the second indicator included bodily injury, rape, kidnapping and fights; and the third indicator included intentional homicide, encounters with cadavers and bones, and robbery and personal injury followed by death. Crimes that occurred outside residences were included, as were robberies and thefts that occurred inside residences. For the construction of the indicators, the total number of each type of crime that occurred from March 2013 to December 2014 was used as the numerator, and the resident population was used as the denominator; the result was then multiplied by 100,000. The unit of analysis for the construction of the indicators was the neighborhood.

The information for the construction of these indicators was obtained from records at the Civil Police of the State of Rio de Janeiro (CPSRJ). The population of individual neighborhoods in the municipality of Rio de Janeiro was also used; the data are available on the website of the Pereira Passos Institute (Instituto Pereira Passos—IPP). To obtain the estimated populations for the year that the data were collected (2014), interpolation was performed using the populations in 2000 and 2010 and the *epolate* command in *Stata 14.0*.

To better capture CV exposure for each individual in their neighborhood, the crime indicators and geocoded residential addresses of the study participants were plotted on a map of the Rio de Janeiro neighborhoods. Buffers (circles with a radius around a point) of 500 m were created around each individual, and the weighted averages of the indicators of crime of all the neighborhoods overlapping with the buffer area were calculated. The weights used were proportional to the overlapping area. Thus, the exposure for each individual was measured not only by the crime rate of their neighborhood but also by the rates of the adjacent neighborhoods for individuals with buffers overlapping more than one neighborhood. All the geoprocessing was performed using *QGIS 3.16* software.

### Outcome variable

CMDs were identified using the validated Brazilian version of the General Health Questionnaire (GHQ-12), which was answered by each adolescent. This instrument consists of 12 questions used to assess psychological distress—non-psychotic mental disorders, defined by anxiety and depression symptoms, inability to deal with ordinary situations and lack of self-confidence. Questions are related to mood, sleep and attention symptoms in the previous 15 days. Each positive response is scored one point, and a score greater than or equal to three was classified as positive for CMDs. The questionnaire is widely used in several countries [20] and has been validated for the Brazilian population using a structured psychiatric interview as the gold standard and has been found to have good psychometric properties, exhibiting sensitivity of 85% and specificity of 79% [21].

### Covariates

Socioeconomic status (SES) was used as a covariate and was constructed based on the Brazilian Economic Classification criterion, which uses information on the possession of goods, the number of bathrooms, having or not having domestic servants, and the education level of the head of the household to establish economic classes, with A corresponding to the highest economic class and E corresponding to the lowest class [22]. All answers were provided by the adolescents.

### Effect modifiers

Sex, age and race were considered possible effect modifiers and were constructed from the responses provided by the adolescents (sex: male or female; race: white, black, brown, Asian and other; race: black, encompassing black and brown, and nonblack, encompassing white, Asian, indigenous and other; and age: 12 to 14 years and 15 to 17 years).

### Statistical analysis

The exposure variables were transformed to their natural logarithm, due to its asymmetrical nature, and then classified as either low or high, with the low level being used as a reference in the regressions. Considering the distribution of crime indicators in the study population and the lack of a consensus regarding their categorization in the literature, we used the median as the cutoff point for division into categories.

Univariate logistic regression modeling was performed, to estimate the gross effect of each independent variable on the dependent variable, followed by adjustment for SES covariates, age, sex, and race. The raw models are not presented herein but may be available upon request. Multivariate logistic regression models were adjusted using the same covariates described above and stratified first by sex, then by age, and finally by race. In the stratified models, the stratification variable was not included as a confounding variable. A statistical significance level of 0.05 was adopted for all analyses, and *Stata* version 14.0 was used for the analyses.

Because the sample was complex and the analyses involved three stages (stratification, selection by clusters and proportional selection probabilities), all analyses considered sample weights. For this purpose, the *svy* module of *Stata* software was used. Thus, in the presentation of the study population, the number of participants from each stratum corresponds to the sample size, and the proportions and respective confidence intervals presented refer to the weighted proportions considering the sample weights, i.e., the total ERICA sample.

## Results

Table 1 provides data for the study population, confounding variables and prevalence rates for outcomes per group. The sample included 52.0% girls, 51% older adolescents [15–17 years] and 56.4% black individuals, and the mean age was 14.57 years (standard deviation (SD) 1.59). The prevalence of CMDs was higher among girls (37.0%) when compared to boys (17.2%), older adolescents (32.0%) when compared to younger ones (23.2%), black individuals (29.2%) when compared to non-black (25.0%), and individuals in economic classes C and D (30.7%) when compared to B (26.6%) and A (23.5%).

**Table 1:** Description of the study population in absolute proportions and expanded relative proportions for the general population and the prevalence of CMDs based on demographic and socioeconomic characteristics. Rio de Janeiro, Brazil (*n* = 1,686)

	Sample description		Prevalence of CMDs
	n	% (95% CI)^a^	% (95% CI)^a^
Sex			
Female	843	52.0 (47.2–56.7)	37.0 (31.6–42.8)
Male	548	48.0 (43.3–52.8)	17.2 (13.1–22.2)
Age			
12 to 14 years	521	51.3 (42.1–60.4)	23.2 (19.6–27.3)
15 to 17 years	870	48.7 (39.6–57.9)	32.0 (26.7–37.7)
Race			
Black (black and brown)	816	56.4 (49.7–62.9)	29.2 (25.4–33.3)
Non-black	554	43.6 (37.1–50.3)	25.0 (20.4–30.2)
Socioeconomic status^b^			
A	93	18.5 (8.0–37.3)	23.5 (11.5–41.9)
B	444	45.0 (38.1–52.1)	26.6 (21.4–32.4)
C and D	405	36.5 (27.2–47.0)	30.7 (24.1–38.1)

^a^Weighted proportions considering the calculation with sample weights

^b^Variable with the highest number of missing data (32.5% missing)

**Fig. 1:**
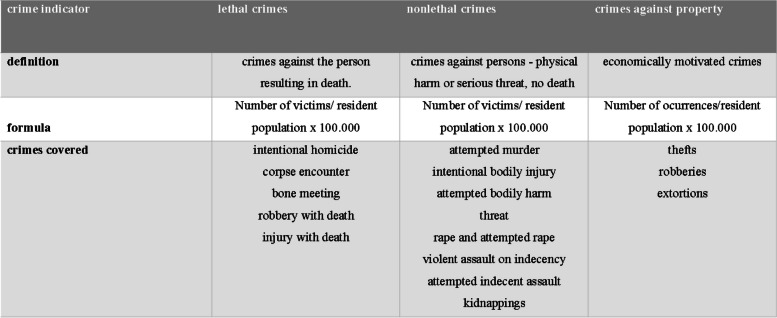
Indicators of community violence – definition, formula and crimes covered

Figure 1 describes the main characteristics of crime indicators, such as its definition, how they were calculated and which crimes they contemplate.

Table 2 presents the minimum and maximum values, mean and medians obtained in the construction of the indicators.

**Table 2:**
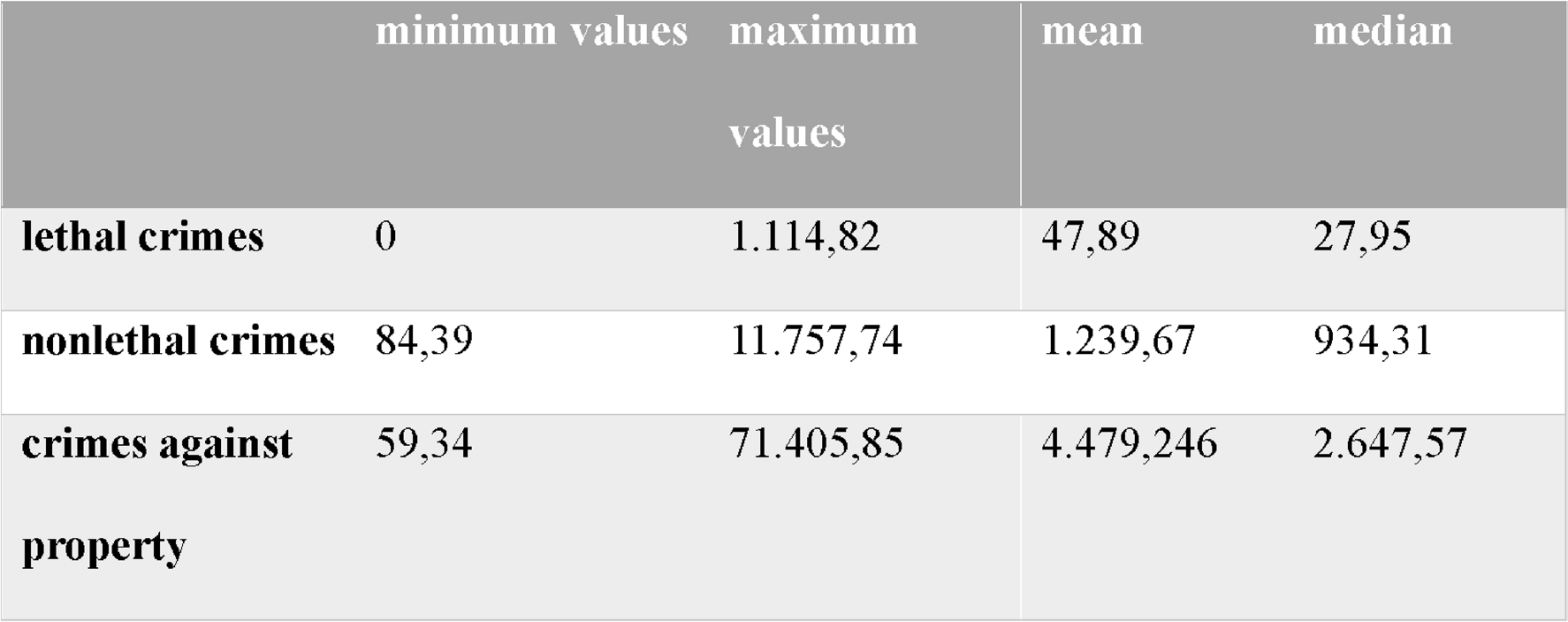
minimum and maximum values, mean and medians of the indicators – rates of lethal crimes against persons, non-lethal crimes against persons and crimes against property

**Fig. 2:**
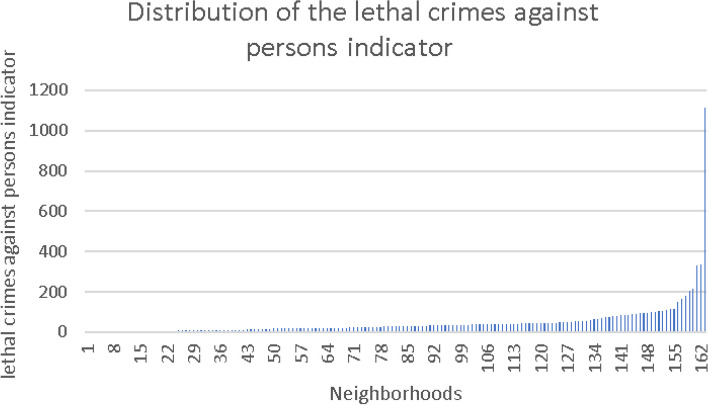
Distribution of the lethal crimes against persons indicator

**Fig. 3:**
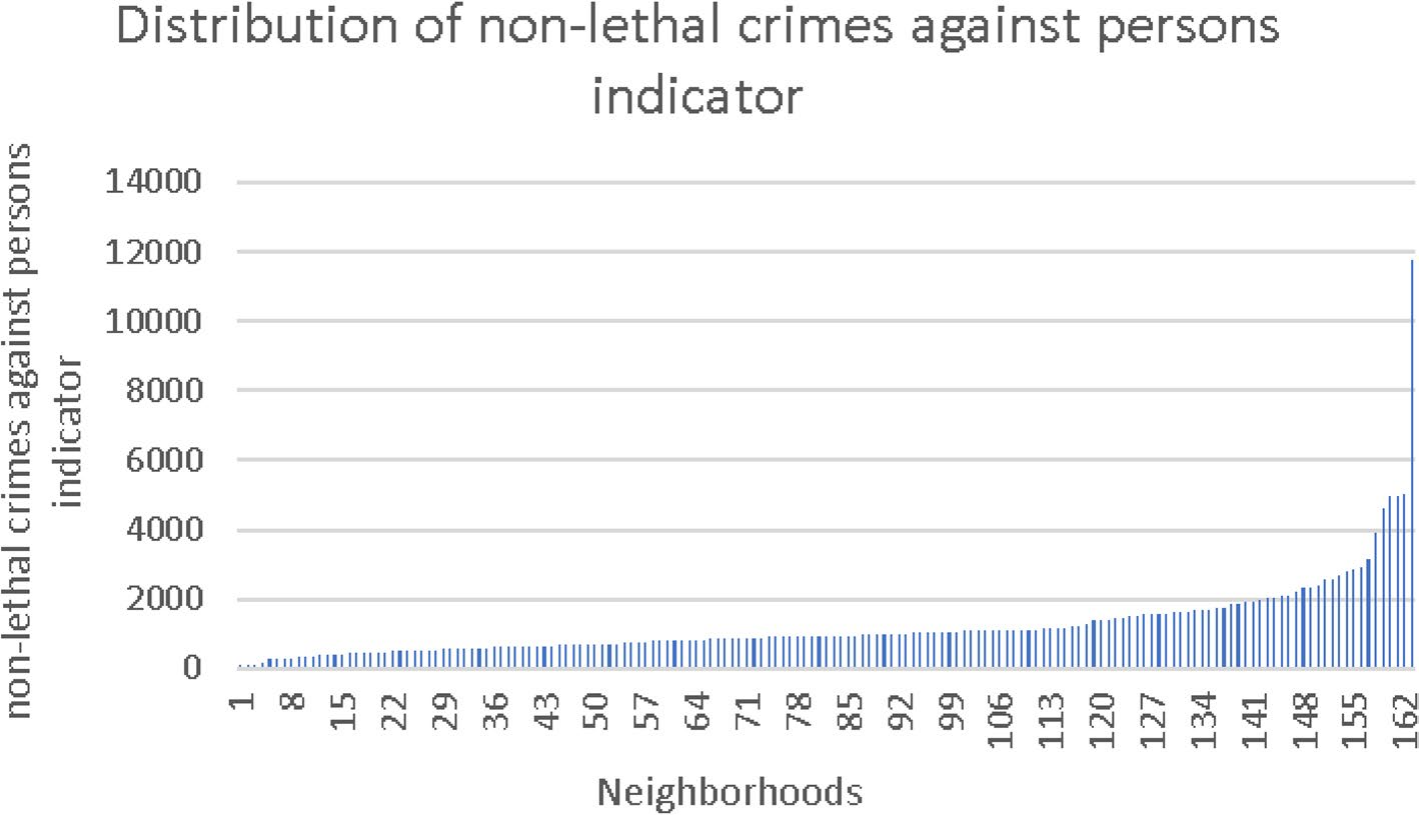
Distribution of non-lethal crimes against persons indicator

**Fig. 4:**
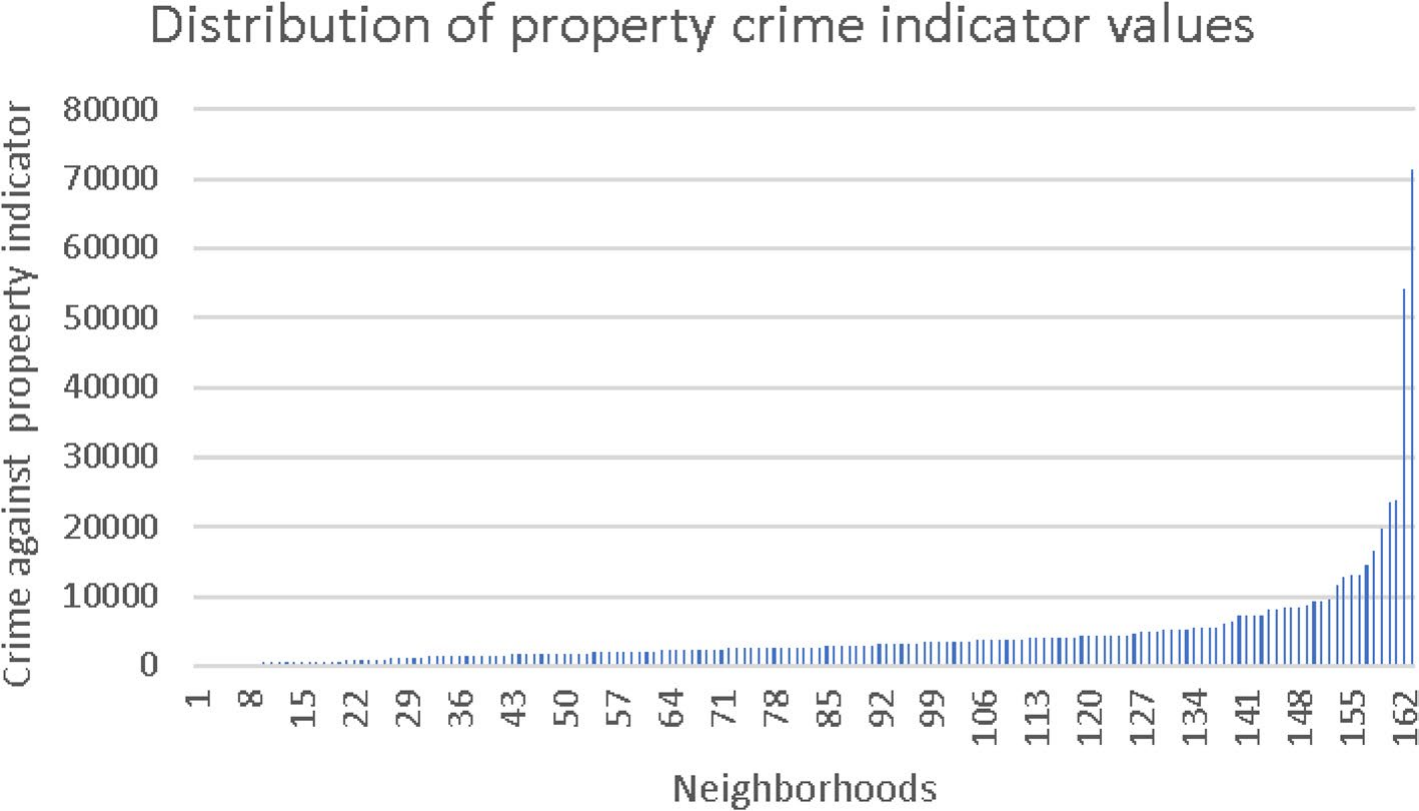
Distribution of the property crimes indicator

Figures 2, 3 and 4 presents the distribution of absolute values of the three crime indicators, before their logarithmic transformation, sorted in ascending order.

The exposure variables were constructed as described before and classified into two levels—low and high, with median as the cutoff point for division into categories. The lower level was used as a reference in the regression models. Sensitivity analyses were performed by operationalizing the indicators into quartiles and quintiles; no benefit was observed.

Table 3 provides the logistic regression results and the odds ratios (ORs) for each crime indicator and CMD outcome. The goodness of fit from all logistic regression models were tested and were adequate. Adolescents living in regions with higher rates of the three types of violence studied herein were more likely to have CMDs, with ORs ranging from 2.33 to 2.99. The greatest effect was observed for crimes against properties. When stratified by sex, girls had a greater magnitude of effect on the association measure when compared to boys, for the three types of violence studied; as well as older adolescents when compared to younger adolescents in the stratification for age; and black individuals compared to nonblack individuals when stratified for race.

**Table 3:** Odds ratios (ORs), upper and lower limits of the confidence interval (LLCIs and ULCIs) and p-values (p) for the logistic regression models adjusted for the CMD outcome

CMD outcome			
Indicators of crime	Crimes against property	Nonlethal crimes	Lethal crimes
	OR (CI)	OR (CI)	OR (CI)
No stratification	2.99 (2.01–4.45) ***	2.48 (1.59–3.88) ***	2.33 (1.65–3.27) ***
Sex			
Female	2.98 (1.71–5.19) **	3.81 (2.20–6.60) ***	3.02(2.06–4.44) ***
Male	1.99 (1.12–3.50) *	0.69 (0.34–1.39)	0.95 (0.43–2.07)
Age			
12 to 14 years	0.65 (0.39–1.06)	0.67 (0.34–1.30)	0.82 (0.53–1.27)
15 to 17 years	5.91 (3.55–9.84) ***	4.76 (3.02–7.48) ***	3.75 (2.16–6.50) ***
Skin color/race			
Black (black and brown)	3.99 (2.24–7.11) **	2.50 (1.44–4.32) *	3.40 (2.10–5.52) ***
Nonblack	0.89 (0.54–1.48)	1.01 (0.59–1.71)	0.73 (0.40–1.30)

^*^ *p* < 0.05; ** *p* < 0.001; *** *p* < 0.0001. Adjusted for socioeconomic status, sex, age and race

## Discussion

The main hypothesis of this study proposes that exposure to community violence acts as a significant risk factor for a higher prevalence of common mental disorders in adolescents. The main results showed that adolescents living in regions with higher rates of the three types of violence were more likely to have CMDs, with ORs ranging from 2.33 to 2.99. The greatest effect was observed for crimes against properties. Since this type of crime is more common and the proportion of people with CMD is fixed across the analyses for all types of crimes (i.e. the same study population), it is reasonable that the proportion of CMD positive people exposed to higher levels of this crime indicator is slightly higher than for other less common types of crime. Thus yielding a slightly higher effect size estimate.

An additional hypothesis was that older, girls, and black adolescents encounter an even greater risk compared to their peers. The results also confirmed this—when stratified by sex, age and race, it becomes clear that the older, the girls, and the black adolescents have higher risks of CMD than their counterparts.

Regarding the first finding – adolescents living in regions with higher rates of violence have higher chances of CMD, no consensus has been established in the literature regarding the association between exposure to CV measured by crime indices and the mental health of adolescents. Two studies conducted in Colombia found positive associations for some outcomes but not for others. The first study, which was performed by Vellez-Gomez, classified regions (communes) based on homicide rates and divided the outcome (depression) into subscales, with a greater likelihood of feelings of ineffectiveness (one of the subscales of the instrument used to measure depression) for adolescents aged 10 to 12 years exposed to high levels of violence; however, the same was not observed for other symptoms of depression [23]. The second study, which was conducted by Cuartas & Roy, evaluated the likelihood of posttraumatic stress disorder and CMDs by comparing adolescents living in high- and low-crime regions through the construction of geospatial indices that considered homicide, and the authors found positive associations between increased exposure to violence and post-traumatic stress disorder (PTSD) and CMDs [24].

On the North American continent, Gepty revealed an association between living in more violent regions and depressive symptoms. However, such conclusions pertained only to adolescents who presented rumination, i.e., a pattern of persistent thoughts related to past events and difficulties in life, as a psychological coping strategy. These authors used as exposure variable the rates of violent crimes, such as homicide, rape and armed robbery, and compare with the rates of nonviolent crimes, such as theft and simple robbery [25]. Grinshteyn studied the association of exposure to violence (homicide, armed robbery and rape) with mental disorders in adolescents using two measures of exposure: direct questions presented to adolescents and criminal statistics. The results indicated a positive relationship only for depression, with a lower magnitude when incorporating the criminal statistics than when incorporating direct victimization suffered by the adolescent [26]. Goldman-Mellor used the same crime rates as a previous study and found no association between living in regions with higher crime rates and psychological distress [27].

The lack of standardized community violence rates presents a challenge in the comparison of studies. Consequently, the formulation of crime rates, encompassing not only homicide rates but also broader crime categories, could offer a potential solution to enhance the comparability of future studies.

Regarding the second finding of this investigation, the heightened risk magnitude for girls in developing CMDs subsequent to exposure to the three types of violence when compared to boys, we can conclude that sex operates as an effect modifier in this association. This result is consistent with some results reported in the literature. When studying the associations between CV and anxiety and depression in adolescents, Bacchini analyzed the difference between sexes and observed that girls had a higher likelihood of developing associated symptoms than boys [28]. Boney-McCoy found similar results for symptoms of sadness and posttraumatic stress [29]. Haj-Yahia and Foster also found that girls who were victims of CV were at a higher risk of developing CMDs; however, this finding was not confirmed for adolescents who were witnesses of violence [30, 31]. Other authors found no differences between sexes [32–34]. As discussed previously, a possible factor that may explain this finding is the sexist culture during the upbringing of children and adolescents, which encourages boys since early childhood not to expose their feelings, while on the other hand, girls are encouraged to be caretakers of the family and home and to not become involved in conflicts and fights [35, 36]. This is a hypothesis for this finding, we understand that more studies are needed to evaluate sex as an effect modifier, as well as studies of qualitative methodology that can study the hypothesis raised in more depth.

Regarding the third finding, that older adolescents (15 to 17 years old) had a higher risk magnitude of having CMDs than younger adolescents (12 to 14 years old) for the three types of violence studied, we concluded that age was an effect modifier in this study, although the literature is inconsistent about his findings. When performing a meta-analysis evaluating the risk of developing mental disorders related to CV exposure, Fowler found a difference regarding to age; children, adolescents, and young adults were considered, and the risk was higher for adolescents (12 to 25 years) than for children (less than 12 years) [9]. Other reviews have shown few studies that analyzed age as an effect modifier in this association, and the results were limited and conflicting, with some suggesting that younger children are more vulnerable to exposure to environmental risks and others suggesting greater vulnerability for older adolescents [5, 10]. Therefore, studies with large samples that analyze a broader age group of adolescents may strengthen the literature in this topic. One possible explanation for our results is that older adolescents spend more time in the city and experience greater exposure to CV than younger adolescents.

Regarding the fourth finding, that black individuals had a higher risk magnitude of having CMDs than non-black individuals after exposure to high levels of CV for the three types of violence studied herein, we concluded that race was an effect modifier in this study. These results are of fundamental importance because despite the large difference between black individuals and white individuals among victims of CV, with young black individuals being predominantly affected [18], few studies have analyzed whether race has a health-modifying effect on the relationship between violence and adolescents’ mental health. A systematic review, carried out by Miliauskas and collaborators, evaluated studies that addressed the association between community violence and internalizing symptoms in adolescents and found that of the 42 analyzed studies, only 2 tested race as a modifier of the effect of the association, and one of them found that black adolescents were more likely to have internalizing symptoms than their white peers [37]. Another study, conducted for Chen and collaborators, found higher levels of depression and delinquency among black adolescents than among white individuals exposed to moderate levels of CV; notably, this effect was not observed with exposure to high levels of violence [38]. The authors attribute this finding to the desensitization hypothesis, where repeated and continuous exposure to CV leads to a decrease in emotional response to it [39]. The same effect occurs for young *Latino* individuals compared to white individuals, suggesting that desensitization is present in other ethnic minorities. In a meta-analysis performed by Fowler [9], race could not be tested as an effect modifier due to an insufficient number of studies with ethnically diverse samples; most of the samples were composed of young black individuals, which complicated such an evaluation, which reinforces the need for studies that encompass heterogeneous samples in terms of race, aiming to examine the extent which this characteristic impacts various health-related aspects. Therefore, the present study included a heterogeneous sample, with national representativeness and a composition of 56.4% black individuals and 43.6% non-black individuals.

Some studies emphasize the experience of black individuals with regard to CV. Borowsky studied a sample of approximately 20,000 adolescents who answered a question about fearing an early death (before age 35), with 25.7% of black individuals answering yes but only 10.2% of white individuals answering yes [40]. When analyzing forms of protection for the black population, Henry studied the effect of maternal support through positive reinforcement of the black culture on protecting the mental health of black adolescents exposed to CV. The results showed that such support was a protective factor for depressive symptoms in the studied sample [41].

An important consideration that can influence our results is the hidden figures not included in crime indices. These hidden figures are understood as the number of crimes not reported to the government. Caetano et al*.* conducted a study using data from the National Household Sample Survey (PNAD) of 2009, collecting information directly from the interviewed individuals regarding being victims of theft, robbery or physical aggression in the last year and whether the incidents were reported to the police. The results indicated a hidden number rate in Brazil of 62.55%; in the state of Rio de Janeiro, the average rate was 63.19% for three types of crime (57.12% for robbery, 69.37% for theft and 65.61% for physical assault). Through probit regression*,* sociodemographic factors were associated with hidden figures for robberies and thefts; the rates were found to be higher for females, people with lower education levels and younger age groups. Therefore, the crime indicators constructed in this study are likely to be significantly higher than those measured through criminal records [42], which means that our indicators are possibly underestimated, as well as the results of the associations, and the reality could be much worse. And as pointed out in this paragraph and the next ones, underreporting is greater in some territories and populations, such as for black residents of favelas and women.

As pointed out by Casseres, *favelas* are territories where the state is almost always unable to guarantee its sovereignty and enforce constitutional order, and this absence is often filled by individuals involved in the illicit drug trade and militias. The author indicates that racism arising from the continuing legacy of the colonial-slave model established in Brazil is currently perpetuated in institutional mechanisms through which the state acts on black people [43]. Therefore, hidden figures can be inferred to be even higher in neighborhoods with favelas. This situation is extremely common given that for this population, police records are even less thorough, which is consistent with the results presented in the study by Caetano et al. [44]*.* The facts presented do not invalidate the results but reinforce the need to evaluate them from the perspective of unequal underreporting, which may represent an even greater effect of CV among black adolescents and favela residents.

Finally, we address the issue of homicides perpetrated by the police. Brazil has a high rate of homicide caused by the state, which is concealed under the guise of acts of resistance, a specific procedure for recording the deaths of civilians resulting from police actions [45]. Thus, homicides committed by police in 2014 were not recorded in the form of intentional homicides and were therefore not included in the crime count for the construction of the lethal crime indicator. Zaccone analyzed archived lawsuits related to records of resistance in the period from 2003 to 2009 in the city of Rio de Janeiro and observed that 75.6% of the records of resistance occurred within favelas; additionally, among the 368 victims, 78% were brown and black, with a mean age of 22 years [46]. Since 2017, the Inter-American Court of Human Rights has ruled that the term “auto de resistance” should no longer be used and should be replaced by personal injury or homicide resulting from police intervention [47]. In 2020, in the state of Rio de Janeiro, 1,245 deaths were attributed to this cause [17]. Therefore, the lethal crime indicator may not reflect the actual total number of deaths in neighborhoods, especially in neighborhoods composed of favelas; these deaths are underestimated, again supporting the hypothesis that the damage to mental health is greater for adolescent black individuals living in favelas, who are the predominant victims of these crimes.

The main strength of this study is the measurement of exposure to CV. Measuring this variable through the creation of crime indices has some advantages, such as the elimination of respondent bias. This bias could have occurred when an adolescent provided information about his or her exposure to violence and mental health because the former can change how he or she perceives the environment and how he or she reacts from an emotional point of view [47]. The classification of these indicators based on the types of crime, which were divided into crimes against property, non lethal crimes, and lethal crimes, can also be highlighted as an advantage because such a strategy allows the investigation of different constructs and intensity levels that may have different effects on adolescents’ mental health. The indicators in this study have the advantage of accounting for the addresses of the sites of the crimes for their construction; homicide rates, for example, were constructed from the victims’ places of residence. Another strength of this study is the location, i.e., the city of Rio de Janeiro, Brazil, which has high rates of violence but few studies of this context.

Losses from and the selectivity of the georeferencing process were limitations because these losses were higher in less urbanized neighborhoods and for adolescents with irregular occupations and a lower SES [48], possibly leading to underestimation of the results. A second limitation is the difficulty of measuring the exposure variable, with the neighborhood serving as the unit of analysis. The municipality of Rio de Janeiro, as well as other municipalities in Brazil, has marked variability in housing conditions within some neighborhoods, with slums and upper middle-class housing in neighboring territories. Thus, the indices for some neighborhoods with high crime rates in a given region may have been mitigated because of less violent regions within the same neighborhood. A third limitation is the relatively high rate of missing data for social class. Many adolescents do not know how to properly inform some of the questions used to create the indicator of social class variable such as education of the head of the family, causing a greater number of missing data. It was not possible to impute this data, which is a limitation of the study. Another limitation in our study is that further neighborhood characteristics, such levels of education, income, and unemployment could be potential confounders, but data was not available. The use of data from different sources, such as the CPSRJ and the Brazilian Institute of Geography and Statistics (IBGE) and IPP, may have generated some degree of inaccuracy when the data were combined; however, this issue was partially circumvented by interpolating population estimates for the same year of data collection, obtaining criminal records for this same period, and then standardizing the rate indices. Last, because this is a cross-sectional study, causality cannot be attributed to the association between CV and CMDs, and this relationship may be bidirectional.

### Final considerations

This study provides important contributions to the field of public health, as it reveals new information on the influence of CV on the mental health of adolescents. Among the main findings are the greater likelihood of CMDs for adolescents living in violent regions who experience both crimes against property and crimes against people, as well as the greater vulnerability of girls, older adolescents and black populations.

Given the high rates of violence globally, which are even higher in low- and middle-income countries, knowing the effects of violence on adolescents is critical to prevent and treat CMDs in this population. There is a pressing need for additional studies that delve into the roles played by race, gender, and age, particularly in regions characterized by elevated violence rates. Such research is essential for identifying the most susceptible demographic groups, thereby contributing to the formulation of effective public policies.

## Data Availability

The datasets used and/or analysed during the current study are available from the corresponding author on reasonable request.

## Abbreviations

CMDs: Common mental disorders
ORs: Odds ratios
CV: Community violence
CPSRJ: Civil Police of the State of Rio de Janeiro
PPI: Pereira Passos Institute
GHQ-12: General Health Questionnaire (GHQ-12)
SES: Socioeconomic status
ERICA: Cardiovascular Risk Study in Adolescents
LLCIs and ULCIs: Lower and upper limits of the confidence interval
PTSD: Post-traumatic stress disorder
PNAD: National Household Sample Survey
IBGE: Brazilian Institute of Geography and Statistics

## Footnotes

1 A special commission was created in 1999 within the state government of Rio de Janeiro to develop a set of indicators of violent crime. The document was produced and signed by the researchers Carlos Henrique Klein, Domício Proença Junior, Eduardo de Azeredo Costa, Galeno Tinoco Ferraz Filho, Ignacio Cano, José Augusto Rodrigues, Paulo Cesar Pinto de Carvalho, Roberto Kant de Lima, and Yolanda Catão.
